# Establishment of Novel and Efficient Methods for Investigating Sexual Reproduction in *Magnaporthe oryzae*

**DOI:** 10.3390/jof11080604

**Published:** 2025-08-20

**Authors:** Yingying Cai, Jing Wang, Muhammad Noman, Zhongna Hao, Zhen Zhang, Haiping Qiu, Rongyao Chai, Yanli Wang, Jiaoyu Wang, Fucheng Lin

**Affiliations:** 1 State Key Laboratory for Quality and Safety of Agro-Products, Key Laboratory of Agricultural Microbiome of Zhejiang Province, Key Laboratory of Biotechnology in Plant Protection of MARA, Institute of Plant Protection and Microbiology, Zhejiang Academy of Agricultural Sciences, Hangzhou 310021, China; caiyy@zaas.ac.cn (Y.C.); wj9311@163.com (J.W.); m.noman@zju.edu.cn (M.N.); haozhongna1999@sina.com (Z.H.); zzhangcn928@sina.com (Z.Z.); qiuhping@163.com (H.Q.); rychai@sina.com (R.C.); ylwang88@aliyun.com (Y.W.); 2 College of Biotechnology and Bioengineering, Zhejiang University of Technology, Hangzhou 310014, China

**Keywords:** conidia mixed mating (CMM), hyphal segments mixed mating (HMM), rice blast, sexual reproduction

## Abstract

Rice blast, caused by *Magnaporthe oryzae*, significantly threatens global rice production. Disease control is complicated by the pathogen’s high genetic diversity, which is driven by heterothallic recombination between opposite mating types that underlies variation. However, mechanisms governing sexual reproduction in this fungus remain poorly characterized, largely due to the absence of reliable methods for scalable ascospore progeny production. In this study, we established two novel mating methods, namely Conidial Mixing Mating (CMM) and Hyphal Segments Mixed Mating (HMM). Both methods employed optimized suspensions (5 × 10^4^ conidia/mL or equivalent hyphal density) mixed at 1:1 ratios, incubated under standardized conditions: 20 °C with a 12 h/12 h photoperiod. We characterized perithecia, asci, and ascospore morphology using fluorescence microscopy, paraffin sectioning, cryo-scanning electron microscopy, and transmission electron microscopy. Furthermore, both methods enabled phenotypic characterization of sexual reproduction-deficient mutants, including Δ*Mopmk1* and Δ*Moopy2*. In conclusion, we established two efficient methods for investigating *M. oryzae* sexual reproduction, providing foundational tools to advance studies of sexual mechanisms, pathogenicity evolution, and genetic variation.

## 1. Introduction

Rice blast caused by *Magnaporthe oryzae* is one of the most devastating crop diseases worldwide, with a profound impact on global rice production [1,2]. Apart from rice, the pathogen can infect more than 50 species of grasses, including barley, wheat, and oats [3]. Breeding and applying resistant rice varieties represents the most effective method of controlling rice blast [4,5]. However, due to the pathogen’s high mutation rate, new highly pathogenic strains emerge frequently and population structures vary continuously, resulting in the resistant rice cultivars losing their resistance after 3–5 years of cultivation [6]. Therefore, the genetic plasticity of the pathogen constitutes a major barrier for the control and prevention of rice blast.

The full life cycle of *M. oryzae* includes both asexual and sexual stages. In the field, the asexual stage is the predominant form for the development and infection of the fungus. The sexual state is inducible in a laboratory environment. However, it has not been found in the field; it has been deemed to occur in nature based on population genetic evidence [7]. For sexual reproduction, two fertile haploid strains of *M. oryzae* are required, which carry different mating type loci, *MAT1-1* and *MAT1-2*, respectively [8,9]. Such a sexual reproduction mode is called heterothallism [10]. On artificial culture media, the mating strains generate spherical perithecia with a long beak, aligning on the junction of the strains. Inside the mature perithecia, numerous elongated and club-shaped asci sprout, with eight ascospores each [11].

In general, sexual reproduction produces more biological variation than asexual reproduction [12,13], because the mating process increases genetic diversity and adaptability through genetic recombination, which allows the sexual progeny to accumulate and utilize genetic information from different parents. Through the elimination of deleterious and harmful mutations during natural selection, sexual progeny can better adapt to changing environments, serving as a crucial pathway driving biological evolution. Studies have indicated that *M. oryzae* originated in Southeast Asia, and based on genetic and ecological evidence, scientists infer that sexual reproduction may occur in some regions [14]. However, direct evidence and detailed mechanisms of sexual reproduction in *M. oryzae* have yet to be elucidated. Over the past few decades, significant progress has been made in studying the production and infection mechanisms of conidiain *M. oryzae* [15,16,17,18,19,20]. Many genes involved in spore production, appressorium formation, infection, and host colonization have been identified and characterized, providing a theoretical foundation for understanding the pathogenicity mechanisms of *M. oryzae* [21,22,23,24]. However, studies on sexual reproduction have been minimal compared to the abundant research findings on the asexual stage of *M. oryzae*. Despite the pivotal role of sexual reproduction in biological evolution, little is known about the impact of *M. oryzae* sexual reproduction on pathogenicity variation and population structure. Therefore, it is imperative to further investigate the process and mechanisms of sexual reproduction in *M. oryzae* to gain a comprehensive understanding of the pathogen’s life cycle and its sources of variation.

One of the key reasons for the lag in sexual reproduction research is the lack of simple and effective methods for inducing sexual reproduction. The traditional method for sexual reproduction induction is to incubate strains with opposite mating types on culture media in a Petri dish [8,11]. At least 20 days post-inoculation (dpi), perithecia develop at the interface between the two fungal strains, forming distinct zonal structures along the colony junction. The traditional method allows strains to produce mature asci, ascospores and perithecia; however, the perithecia zone formed on the media is narrow and limited, usually only a few millimeters wide (2–3 mm) [9,25]. This results in a severe shortage of sexual progeny. For researchers, this limited number of sexual progeny greatly hinders further research, especially at the molecular level. For example, experiments such as RNA sequencing, proteomics, and metabolomics analysis require a large number of samples, which cannot be satisfied by the limited number of sexual progeny samples provided by traditional methods.

In light of the limitations of the traditional cross-mating method (TCM) in studying sexual reproduction of *M. oryzae*, such as the long cycle time and low efficiency of producing sexual progeny, two more efficient and stable mating methods, conidia mixed mating (CMM) and hyphal segments mixed mating (HMM), were established in this study. These two new methods showed significant advantages in the amount, generating time, and uniformity of production of the sexual structures, perithecia, and ascospores.

Through condition optimization experiments, we found that temperature, light, and concentration of conidial suspension were important factors affecting perithecium formation. Perithecium development exhibited optimal progression at 20 °C, with ascospore production requiring photoperiod induction. Notably, insufficient spore concentrations significantly impaired sexual structure formation. More importantly, the sexual progeny produced by these two methods are sufficient in both quantity and quality to meet the needs of subsequent experiments such as RNA extraction, reverse transcriptase-quantitative PCR (RT-qPCR), and transcriptome analysis. We observed the sexual reproductive structures formed by the CMM and HMM methods using fluorescence microscopy, paraffin sections, cryo-scanning electron microscopy, and transmission electron microscopy. Furthermore, the CMM and HMM methods enabled more rapid and accurate determination of mutant strain sexual reproductive capacity. These results lay a solid foundation for in-depth research on the genetic mechanisms, pathogenicity, and control strategies of *M. oryzae*.

## 2. Materials and Methods

### 2.1. Fungal Strains and Culture Conditions

The *M. oryzae* strains Guy11 (*MAT1-1*), TH16 (*MAT1-1*), 2539 (*MAT1-2*), 70-15 (*MAT1-2*), and TH3 (*MAT1-2*) were cultured on complete medium (CM) according to routine procedures for mycelia development and conidia preparation [15]. These strains were preserved by the filter paper sheet method for long-term storage.

### 2.2. Traditional Cross-Mating Method (TCM)

**Figure 1:**
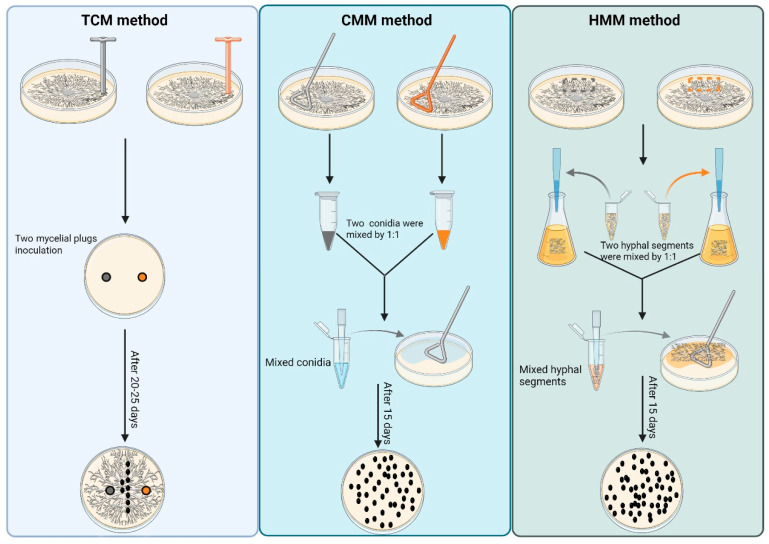
Schematic comparison of three methods for mass production of sexual progeny in *M. oryzae.* TCM method: Opposing mating-type strains were co-cultured on CM plates at 28 °C under 12 h/12 h photoperiod for 5 d. Cultures were subsequently transferred to 20 °C with continuous illumination for 15–20 d to induce perithecia formation. CMM method: Conidial suspensions (5 × 10^4^ conidia/mL) from complementary mating types were mixed in equal volumes (100 μL each). The combined suspension was spread on oatmeal agar (OMA) and incubated at 20 °C under constant light for 15 d. HMM method: Opposing mating types were separately cultured in liquid CM at 25 °C with agitation (150 rpm) for 48 h. Hyphal masses (100 mg each) were physically disrupted using high-frequency oscillation (60 Hz, 90 s). The fragmented hyphae (400 μL aliquots) were co-inoculated on OMA plates and maintained at 20 °C under continuous light for 20 d.

The traditional method for mating was performed as described previously with minor modifications [26]. Opposite mating type *M. oryzae* strains were co-inoculated on 9 cm Oatmeal Agar (OMA) plates and maintained at 28 °C with a 12 h/12 h light/dark cycle for 5 d to allow the colonies to make contact with each other. Subsequently, the culture plates were transferred to 20 °C with full light for 15 to 20 d (Figure 1, Table 1).

### 2.3. Conidia Mixed Mating Method (CMM)

The *M. oryzae* strains were cultured on 9 cm CM plates at 28 °C with a 12 h/12 h light/dark cycle. At 9 dpi, the conidia were harvested with a sterilized applicator stick, filtered through triple filter paper into a 1.5 mL centrifuge tube, and the conidial suspension was adjusted to 1 × 10^5^ conidia/mL. A measure of 100 μL of conidial suspension from each of the strains with opposite mating types was mixed and evenly distributed onto the OMA plates with a sterilized applicator stick. The inoculated plates were then incubated at 20 °C under full light conditions for 15 d (Table 1). Each treatment was repeated in triplicate. The development of sexual structures was recorded and observed under a microscope (Figure 1).

### 2.4. Hyphal Segments Mixed Mating Method (HMM)

Hyphae of opposite mating types were scraped from the plates into liquid CM and incubated at 25 °C and 150 rpm/min on a shaker for 2 d. Subsequently, the hyphae in the liquid CM were filtered with triple filter paper under an ultra-clean bench and pressed dry with absorbent paper to obtain hyphal segments. Subsequently, hyphae of opposite mating types (100 mg of each) were combined into a 2 mL centrifuge tube. Afterwards, sterilized 3 mm steel beads and 400 μL of sterile ddH_2_O were added to this centrifuge tube. The hyphae were broken into small segments by shaking on an oscillating crusher at a frequency of 60 Hz for 90 s. Then, 400 μL of the hyphal segments mixture was evenly applied to the OMA plates and incubated at 20 °C for 15 d under full light conditions (Table 1). Each set of experiments was repeated in triplicate, and the formed sexual structures were observed under the microscope (Figure 1).

### 2.5. Observation of Sexual Structures

The number and structures of perithecia, asci, and ascospores were observed under a stereoscope, a fluorescent microscope (Zeiss), using Calcofluor White (CFW) staining, and paraffin sections with PAS (Periodic acid–Schiff) staining. The ultra-structure of sexual progeny was examined using scanning electron microscope (SEM) and transmission electron microscope (TEM).

The perithecia generated on OMA plates were picked up with a sterilized inoculation needle and placed on a glass slide. A measure of 10 μL CFW staining solution (50 μg/mL) was added to the perithecia, a coverslip was placed over the sample, and perithecia were crushed by gently pressing the coverslip with the pipette tip, releasing the asci and ascospores. After staining for 5 min, the slide was observed under a confocal fluorescence microscope (Zeiss, Jena, Germany) with an excitation wavelength of 405 nm and emission wavelength of 410–480 nm.

The 3 mm culture blocks forming the perithecia were excised with a sterilized knife to prepare paraffin sections using a standard method. Paraffin sections were stained with PAS and observed under a light microscope.

For SEM, the 3 mm culture blocks with perithecia were rapidly frozen in liquid nitrogen and observed under a cryo-environmental SEM (Hitachi, Tokyo, Japan). For TEM, the perithecia were picked up as previously described, fixed with glutaraldehyde, and examined under a TEM (Hitachi, Tokyo, Japan).

### 2.6. RNA Extraction and RT-qPCR

Approximately 100 mg of perithecia were collected from OMA medium of 10 d, 15 d, and 20 d HMM and CMM cultures, using a sterilized knife. Total RNA was extracted using an RNA purification kit (Tiangen, Beijing, China). The quality and quantity of the RNA samples were assessed by gel electrophoresis and spectrophotometric measurement of absorbance at 260 nm. The extracted RNA was used for reverse transcription to generate cDNA using the PrimerScript RT reagent kit (TaKaRa, RR047B, Kusatsu, Japan), which served as a template in RT-qPCR experiments. Further details on data processing can be found in the article by Qiu et al. [27]. Primers for detecting gene expression are shown in Table S1 in the Supplemental Materials.

### 2.7. Condition Optimization for Sexual Reproduction

To investigate the environmental conditions affecting the sexual reproduction of *M. oryzae* and optimize the generation of perithecia and ascospores in the CMM and HMM methods, we compared the formation efficiency of sexual structures under different conditions: 8 temperature points (12, 15, 17, 20, 23, 25 and 27 °C), 3 light cycles (24 h darkness, 12 h/12 h light–dark alternation and 24 h light), and 6 conidial concentrations (5 × 10^3^/mL, 1 × 10^4^/mL, 2 × 10^4^/mL, 4 × 10^4^/mL, 8 × 10^4^/mL and 1 × 10^5^/mL). Guy11/TH3 and TH16/TH3 strain combinations were used in this experiment.

### 2.8. Phosphorylation Assays

Total proteins were extracted from liquid CM-cultured mycelium of *M. oryzae* strains using TCA-acetone precipitation approaches [28,29]. The phosphorylated MoPmk1 level was assessed by Western blotting. Anti-phospho-p44/42 MAPK antibody (Cell Signaling Technology, Danvers, MA, USA) and anti-p44/42 MAPK antibody (Santa Cruz Biotechnology, Dallas, TX, USA) were used to detect the phosphorylated and non-phosphorylated forms of MoPmk1. Anti-GAPDH antibody (HUABIO, Hangzhou, China) was used for the detection of GAPDH. Band intensities were measured with ImageJ software (version 1.54p).

## 3. Results

### 3.1. Mass Perithecia Generated via CMM and HMM Methods

**Figure 2:**
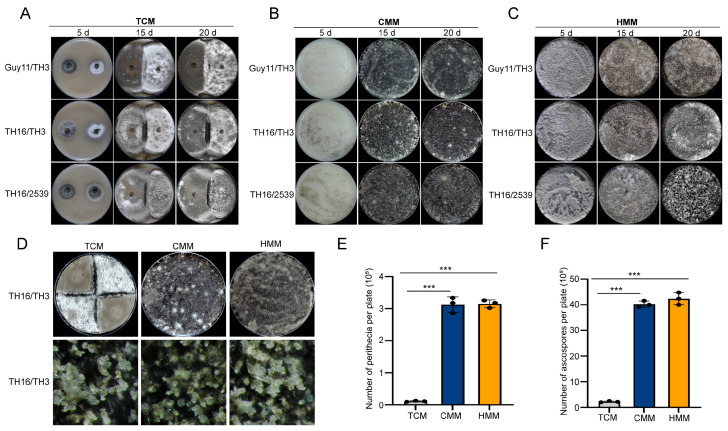
Inoculation of strain combinations Guy11/TH3, TH16/TH3, and TH16/2539 using TCM, CMM, and HMM methods. (**A**) Three different strain combinations were inoculated on OMA plates using the TCM method. (**B**) Three different strain combinations were inoculated on OMA plates using the CMM method. (**C**) Three different strain combinations were inoculated on OMA plates using the HMM method. (**D**) TH16/TH3 strain combination was inoculated on OMA plates using the three methods. (**E**) Statistical analysis of the number of perithecia produced through inoculation using the three methods. (**F**) Statistical analysis of the number of ascospores produced through inoculation using the three mating methods. Significantly different datasets are labelled with asterisks (***) at *p*-value ≤ 0.001).

Different combinations of strains were inoculated on OMA plates by TCM, CMM, and HMM methods. Compared to the two mycelial plugs inoculated using the TCM method, which had not yet made contact with each other after 5 d (Figure 2A), the conidia hyphal segments inoculated with the CMM and HMM methods germinated rapidly, and the newborn hyphae could be observed at 2 dpi, at which time the hyphae of the two strains had begun to come into contact with each other. The hyphae continued to grow, completely covering the surface of the medium at 5 dpi. The number and size of the perithecia gradually increased, ultimately achieving a dense distribution across the medium’s surface by 15 d (Figure 2B,C).

Under a stereomicroscope, there were no significant differences in size, distribution, density, and morphology between the perithecia formed by the two mating methods (CMM and HMM) and those formed by the TCM method under the same conditions (Figure 2D). The number of perithecia and ascospores formed by 15 dpi in the CMM and HMM methods and 30 dpi in the TCM method was counted. Results showed that the number of perithecia produced on each Petri dish using the mixed-culture mating methods was nearly 30 times higher than that of the TCM method, and the number of ascospores produced was nearly 20 times higher than that of the TCM method (Figure 2E,F). There were no significant differences in the number of perithecia and ascospores produced between the CMM and HMM methods. Compared to the TCM method, the CMM and HMM methods exhibited advantages in terms of the number of perithecia and ascospores produced.

### 3.2. Condition Optimization of CMM and HMM Methods

**Figure 3:**
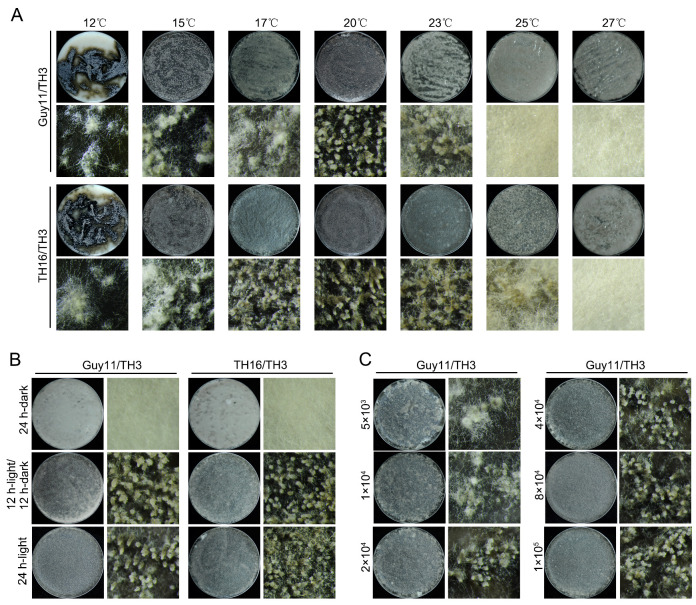
Conditional optimization of the CMM method. (**A**) The efficiency of Guy11/TH3 and TH16/TH3 strain combinations in perithecia formation at different temperatures. (**B**) The efficiency of Guy11/TH3 and TH16/TH3 strain combinations in perithecia formation at different light exposure conditions. (**C**) The efficiency of Guy11/TH3 strain combination in perithecia formation at different concentrations of conidial suspension (conidia/mL).

To optimize the conditions for CMM and HMM methods and investigate the environmental factors influencing sexual reproduction, we compared the efficiency of sexual progeny formation in *M. oryzae* strains at different temperatures, light regimes, and conidial suspension concentrations. The results indicated that all three factors were crucial for perithecium formation. For the Guy11/TH3 or TH16/TH3 combinations, the optimal temperature for perithecium formation was 20 °C, but there were differences in temperature adaptation between the two strain combinations. Excessively low or high temperatures were detrimental to the perithecium formation (Figure 3A). Under complete darkness, the strains were unable to form sexual structures. The 12 h/12 h light/dark cycles and continuous light conditions stimulated the production of perithecia by the two mating type combinations, with no significant difference in morphology or quantity, indicating that light exposure of a certain duration could induce sexual reproduction without necessarily requiring 24 h continuous light (Figure 3B). Excessively low spore concentrations were unfavorable for perithecium formation. When the spore concentration increased to 5 × 10^4^ conidia/mL, the Guy11/TH3 combination could effectively form sexual progeny. Further increases in spore concentration had no significant effect on enhancing the number of perithecia (Figure 3C).

### 3.3. High-Purity RNA Extraction and Verification

**Figure 4:**
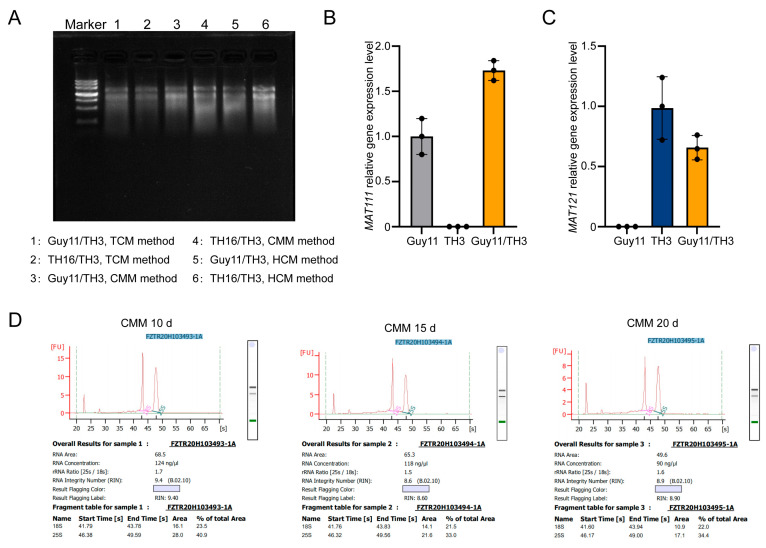
Extraction and quality assessment of RNA from perithecia. (**A**) Electrophoretic verification of RNA extracted from perithecia obtained via TCM, CMM, and HMM methods. (**B**) Relative expression levels of MAT1-1-1 in perithecia produced by inoculation of the Guy11/TH3 strain combination using the CMM method, as detected by RT-qPCR. (**C**) Relative expression levels of MAT1-2-1 in perithecia produced by inoculation of the Guy11/TH3 strain combination using the HMM method, as detected by RT-qPCR. (**D**) Concentration and purity of RNA extracted from perithecia produced using CMM at 10 d, 15 d, and 20 d, as assessed using the Agilent 2100 Bioanalyzer (Santa Clara, CA, USA).

To evaluate whether the CMM and HMM methods could yield material suitable for high-purity RNA extraction, we extracted RNA from perithecia obtained by the CMM, HMM, and TCM methods. Subsequently, gel electrophoresis was used to assess the purity and integrity of the extracted RNA (Figure 4A). The results showed that all three methods yielded high-quality RNA from the sexual progeny of *M. oryzae*. Subsequently, the RNA extracted from the perithecia was reverse-transcribed into cDNA. Further validation of the effectiveness of the CMM and HMM methods was conducted using RT-qPCR (Figure 4B,C). The expression analysis revealed distinct distribution patterns between the two mating-type genes. Specific *MAT111* expression signals were detected in Guy11/TH3 hybrid perithecia via the CMM and in Guy11 conidia, whereas no detectable expression signals were observed in TH3 conidia. Conversely, *MAT121* expression was exclusively observed in Guy11/TH3 hybrid perithecia via the HMM and in TH3 hyphae, with no corresponding expression signals detected in Guy11 hyphae. Additionally, RNA was extracted from perithecia formed at 10, 15, and 20 dpi using the CMM for transcriptome analysis. As shown in Figure 4D, the RNA concentrations of the three RNA samples were 124 ng/μL, 118 ng/μL, and 90 ng/μL, respectively, all meeting the RNA concentration requirement (>60 ng/μL) for transcriptome experiments. The RNA integrity numbers (RINs) of the three RNA samples were 9.4, 8.6, and 8.9, respectively, exceeding the RIN threshold of 6.5 required for transcriptome analysis. These results suggest that the quantity of sexual progeny generated by the CMM and HMM methods is sufficient for RNA extraction, RT-qPCR, and transcriptome analysis.

**Figure 5:**
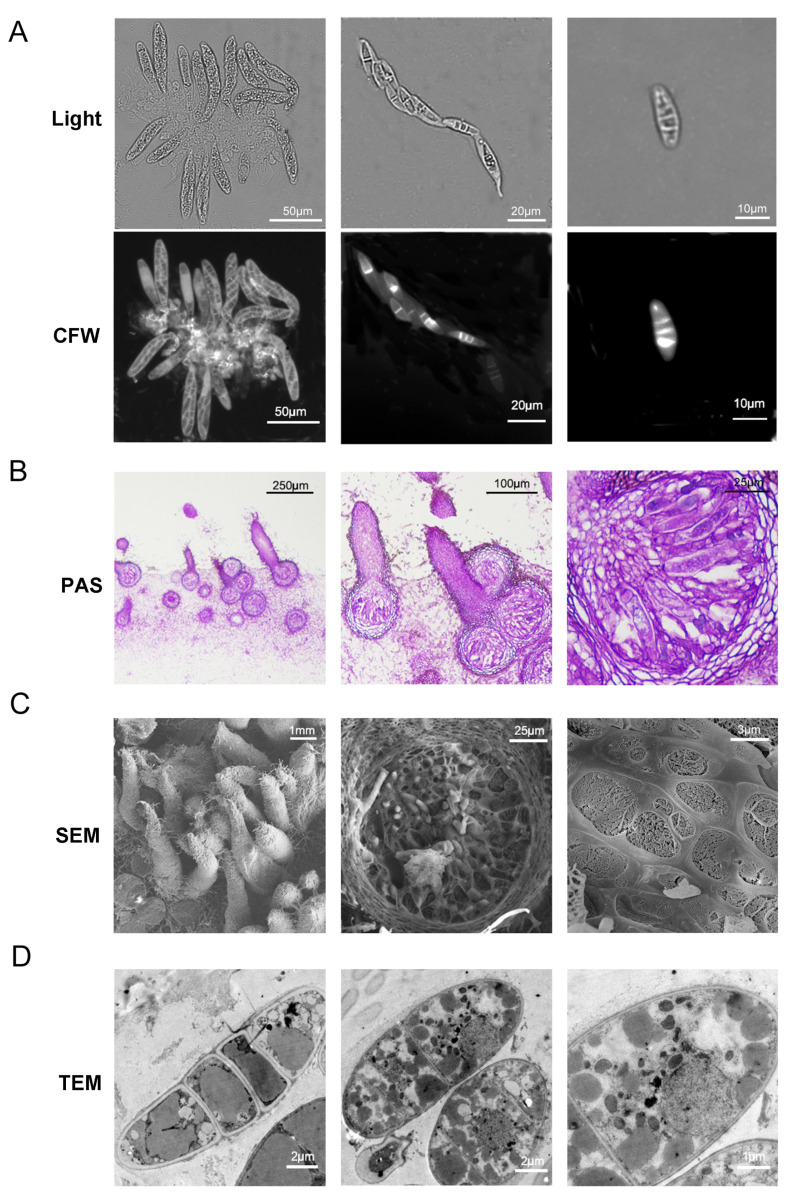
Observation of sexual reproductive structures via multiple microscopy techniques. (**A**) Visualization of ascospores and asci containing eight well-arranged ascospores within perithecia using CFW. (**B**) Morphological characterization of asci in paraffin-sectioned specimens with PAS staining. (**C**) Ultrastructural analysis of perithecia via cryo-SEM. (**D**) High-resolution imaging of ascospore internal ultrastructure using TEM. The rightmost panel is a magnified view of a region within the middle panel.

To better understand the structure of the sexual progeny of *M. oryzae* and verify the maturity of perithecia formed by both CMM and HMM methods, we observed the structure of perithecia using CFW staining. Under a fluorescence microscope, perithecia formed by 15 dpi contained a large number of asci, each of which harbored eight uniformly arranged and evenly sized ascospores (Figure 5A), consistent with the previously reported structure of perithecia [11]. Paraffin sections of the perithecia and observation with PAS staining showed that ascospores were regularly arranged within the inner wall of the asci (Figure 5B). In addition, paraffin sections also revealed that the spherical portion of the perithecium was embedded within hyphal tissue, rather than within the interior of the medium. Further observations using cryo-SEM showed a dense distribution of perithecial beaks on the surface of fungal tissue. On randomly fractured surfaces, perithecia, ascospores, and ascus walls were observable (Figure 5C). The internal structure of perithecia, as well as organelles such as nuclei and mitochondria, could be clearly observed by TEM. Mature ascospores generally contained four cells, each of which typically contained a large oil globule (Figure 5D). Interestingly, the cells of ascospores could be easily separated, and TEM analysis revealed that the intervals between ascospore cells were double-layered, with each cell forming a complete cell wall. These results suggest that the differentiation mode of ascospores may be entirely distinct from that of conidia (Figure 5D).

### 3.4. The CMM and HMM Methods Generate More Perithecia

**Figure 6:**
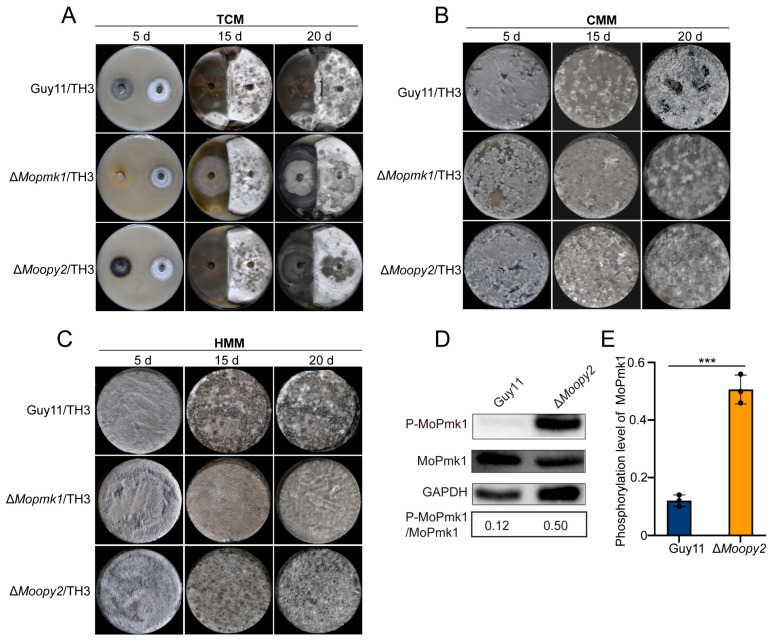
Inoculation of wild-type (Guy11) and mutant strain combinations using TCM, CMM, and HMM methods. (**A**) Guy11/TH3, Δ*Mopmk1*/TH3, and Δ*Moopy2*/TH3 strain combinations were inoculated on OMA plates using the TCM method. (**B**) Guy11/TH3, Δ*Mopmk1*/TH3, and Δ*Moopy2*/TH3 strain combinations were inoculated on OMA plates using the CMM method. (**C**) Guy11/TH3, Δ*Mopmk1*/TH3, and Δ*Moopy2*/TH3 strain combinations were inoculated on OMA plates using the HMM method. (**D**) Phosphorylation levels of MoPmk1 in Guy11 and Δ*Moopy2* mutants. (**E**) Statistical analysis of MoPmk1 phosphorylation levels in Guy11 and Δ*Moopy2* mutants. Significantly different datasets are labelled with asterisks (***) at *p*-value ≤ 0.001).

In *Saccharomyces cerevisiae*, Fus3 MAPKs are crucial components of the MAPK signaling pathway, participating in the mating and fusion processes, which are key steps in sexual reproduction [30]. MoPmk1 is the homolog of Fus3 in *M. oryzae.* Previous studies have shown that MoOpy2 interacts with the scaffold protein Mst50, which plays a pivotal role in the Pmk1 MAPK signaling pathway [17,31,32]. Guy11/TH3, Δ*Mopmk1*/TH3, and Δ*Moopy2*/TH3 strain combinations were inoculated on OMA plates by the TCM method, and only Guy11/TH3 produced black perithecia at the interface of the two strains, while no perithecia were formed in the combinations of Δ*Mopmk1*/TH3 and Δ*Moopy2*/TH3 at 20 dpi (Figure 6A). To further confirm the roles of MoPmk1 and MoOpy2 in sexual reproduction, Guy11/TH3, Δ*Mopmk1*/TH3, and Δ*Moopy2*/TH3 were inoculated using the CMM and HMM methods. At 20 dpi, all three combinations produced perithecia using the CMM method, but the number of perithecia produced by Δ*Mopmk1*/TH3 and Δ*Moopy2*/TH3 was less than that produced by Guy11/TH3 (Figure 6B). The same strains were inoculated by the HMM method, perithecia were produced at 15 dpi (Figure 6C). These results suggest that the CMM and HMM methods allow mutant strains with reduced sexual reproduction capacity to produce sexual progeny more quickly compared to the TCM method.

To further investigate whether MoOpy2 regulates the Pmk1 MAPK signaling pathway, we examined the MoPmk1 phosphorylation levels in Guy11 and the Δ*Moopy2* mutant, respectively. As shown in Figure 6D,E, the level of MoPmk1 phosphorylation in the Δ*Moopy2* mutant was approximately 4.17-fold higher than that in Guy11, suggesting that aberrant activation of MoPmk1 in the *MoOPY2* deletion mutant may be responsible for the defect in sexual reproduction.

## 4. Discussion

In this study, two new mating methods were established to address the critical limitation of efficient methods to produce a large number of sexual progeny in the study of sexual reproduction of *M. oryzae*: the CMM method and the HMM method. These two new methods have significantly improved the efficiency of producing perithecia and ascospores, thereby adequately fulfilling the prerequisites for subsequent experimental procedures, including RNA extraction, RT-qPCR, and transcriptome analysis.

Compared to the TCM method, the CMM and HMM methods exhibit significant advantages in terms of producing perithecia and ascospores. Under identical culture conditions, the number of perithecia per Petri dish using the CMM and HMM methods was nearly 30 times higher than that of the TCM method, and the number of ascospores produced was nearly 20 times higher than that of the TCM method. These quantitative improvements provided a sufficient sample size for subsequent experiments. Furthermore, through conditional optimization experiments, this study identified key factors influencing perithecium formation: temperature, light exposure, and concentration of conidial suspension. The optimal temperature was 20 °C, and sexual reproduction could be induced under specific light exposure conditions, while insufficient spore concentrations significantly impaired the perithecium formation. These findings contribute to optimizing the experimental conditions for the CMM and HMM methods and offer a valuable reference for future research. By precisely controlling these parameters, the efficiency of perithecium and ascospore production can be further improved to meet the needs of diverse experimental requirements.

We also successfully extracted high-purity RNA from the perithecia produced by the CMM and HMM methods, as verified through electrophoresis and RT-qPCR. These results demonstrated that the sexual progeny produced by the CMM and HMM methods were sufficiently abundant and of high quality to satisfy the needs of subsequent experiments such as RNA extraction, RT-qPCR, and transcriptome analysis.

Using fluorescence microscopy, paraffin sections, cryo-SEM, and TEM, the structural characteristics of perithecia, asci, and ascospores were observed. Beyond validating the CMM and HMM methods, these observations furnish valuable morphological data crucial for probing the underlying microscopic mechanisms of sexual reproduction in *M. oryzae*. In particular, the observations under TEM revealed the internal structure of ascospores and the distribution characteristics of organelles, which are significant for understanding the differentiation and development processes of ascospores.

Additionally, the CMM and HMM methods enabled mutant strains with diminished sexual reproductive ability, such as Δ*Mopmk1* and Δ*Moopy2*, to produce sexual progeny more efficiently, which allowed for a more precise determination of the specific roles of these genes in the sexual reproduction process. The mitogen-activated protein kinase (MAPK) cascade plays a pivotal role in the response of plant pathogens to host and environmental signals. The MAPK signaling pathway operates through sequential phosphorylation via MAPKKKs (Ste11, Ssk2/22, and Bck1/2), MAPKKs (Ste7, Pbs2, and Mkk1/2), and MAPKs (Fus3/Kss1, Slt2, and Hog1) [33]. Phosphorylated MAP kinases regulate the expression of genes in response to external environmental signals by activating a series of downstream transcription factors, thereby controlling cellular functions [34]. In filamentous fungi, the Fus3 homolog kinase typically participates in the regulation of sexual reproduction and filamentous growth, a role that has been demonstrated in *Aspergillus nidulans* [35], *Fusarium graminearum* [36], *Aspergillus fumigatus* [37], and *Aspergillus cristatus* [38]. In this study, we found that MoPmk1 (Fus3 homolog in *M. oryzae*) and MoOpy2 play crucial roles in the sexual reproduction of *M. oryzae*. The Δ*pmk1*/TH3 and Δ*Moopy2*/TH3 mutant strain combinations failed to form perithecia by TCM method. However, both mutant combinations produced perithecia using the CMM and HMM method. This indicates that closer cellular contact in liquid/solid media allows TH3 wild-type alleles to partially rescue the sexual defects of Δ*Mopmk1* and Δ*Moopy2* mutants. These results collectively suggest that MoPmk1 and MoOpy2 may affect the sexual reproduction process by regulating the Pmk1 MAPK signaling pathway. Moreover, the abnormally elevated level of MoPmk1 phosphorylation in the Δ*Moopy2* mutant may contribute to the defective sexual reproduction. In *M. oryzae*, the Pmk1 MAPK signaling pathway is involved not only in sexual reproduction but also in appressorium formation, penetration peg invasion, and hyphal infectious growth [34]. Previous studies have reported that the five key genes *MAT111*, *MAT112*, *MAT113*, *MAT121*, and *MAT122*, which participate in sexual reproduction, are not essential for hyphal growth, asexual reproduction, and pathogenicity in *M. oryzae* [11]. This suggests that sexual reproduction and pathogenicity may be two independent pathways. However, the regulatory factors controlling these two pathways remain unclear and require further in-depth investigation.

## 5. Conclusions

In conclusion, the newly established CMM and HMM methods provide a novel approach to the study of sexual reproduction in *M. oryzae*. These two methods not only improve the efficiency of perithecia and ascospores but also broaden the scope of investigation, facilitating phenotypic characterization of mutants with impaired sexual reproductive capacity. Conditional optimization and observation of microstructures further revealed the molecular mechanisms and influencing factors of sexual reproduction in *M. oryzae*. These findings offer significant theoretical support and practical guidance for deepening the understanding of the genetic mechanisms, pathogenic processes, and control strategies of *M. oryzae*. In the future, we will continue to leverage these methods to delve deeper into the sexual reproductive process of *M. oryzae*, aiming to provide novel insights and methodologies for the prevention and control of rice blast.

## Figures and Tables

**Table 1:** The optimal conditions for the formation of perithecia.

	Temperature	Light Exposure	Conidium Density
Optimal conditions	20 °C	24 h light exposure	5 × 10^4^ conidia/mL

## Data Availability

Datasets used or analyzed during the current study are available in the manuscript.

## Supplementary Materials

The following supporting information can be downloaded at: https://www.mdpi.com/article/10.3390/jof11080604/s1, Table S1: Primers used in this study.
