# Supplementary material for: Establishment of Novel and Efficient Methods for Investigating Sexual Reproduction in Magnaporthe oryzae
Source: J Fungi (Basel). 2025 Aug 20;11(8):604. doi: 10.3390/jof11080604 (PMC12387719; doi:10.3390/jof11080604)
Supplement: Supplementary file 1 [file jof-11-00604-s001.zip › jof-3739130-supplementary.pdf]

Table S1 Primers used in this study

Primer name	Primer sequence (5'-3')
MAT1-1-F1	GCTGGCATCTTTCAGGATAGACCG
MAT1-1 R1	GTGTTTCCGGGTGACCATGACCTT
MAT1-2-F1	AGGGTCACGGATTTGTCTG
MAT1-2-R1	TTGCTTTGCTCGGCTTCG
